# Missouri K-12 school collection and reporting of school-based syndromic surveillance data: a cross sectional study

**DOI:** 10.1186/s12889-016-2771-0

**Published:** 2016-02-01

**Authors:** Terri Rebmann, Allison K. Kunerth, Alan Zelicoff, Michael B. Elliott, Harper F. Wieldt

**Affiliations:** 1 Department of Environmental and Occupational Health, Institute for Biosecurity, Saint Louis University, College for Public Health & Social Justice, 3545 Lafayette Avenue Room 463, Saint Louis, MO 63104 USA; 2 Department of Biostatistics, Saint Louis University, College for Public Health & Social Justice, Saint Louis, MO USA; 3 Villa Duchesne High School, St. Louis, MO USA

**Keywords:** Pandemic, Emerging infectious disease, Bioterrorism, Public Health

## Abstract

**Background:**

School participation in collecting and reporting syndromic surveillance (SS) data to public health officials and school nurses’ attitudes regarding SS have not been assessed.

**Methods:**

An online survey was sent to Missouri Association of School Nurses members during the 2013/2014 school year to assess whether K-12 schools were collecting and reporting SS data. Z-scores were used to assess collection versus reporting of SS indicators. Logistic regressions were used to describe factors predicting nurses’ collection and reporting of SS indicators: all-cause absenteeism, influenza-like illness and gastrointestinal illness. Univariate predictors were assessed with Chi-Squares.

**Results:**

In total, 133 school nurses participated (33.6 % response rate). Almost all (90.2 %, *n* = 120) collect at least one SS indicator; half (49.6 %, *n* = 66) report at least one. Schools are collecting more SS data than they are reporting to the health department (*p* < .05 for all comparisons). Determinants of school nurses’ collection of SS data included perceived administrative support, and knowledge of collecting and analyzing SS data. The strongest predictive factors for reporting SS data were the perception that the health department was interested in SS data and being approached by the health department to collect SS data.

**Conclusion:**

Schools are collecting SS indicators at a relatively high rate, yet less than half of the data is reported to public health officials. Findings from this study indicate that public health officials can increase access to school-based SS data by approaching schools about collecting and reporting this important data.

## Background

Large-scale biological events, such as bioterrorism attacks or a pandemic, can threaten the health of citizens worldwide, particularly if the event involves a communicable disease. For example, in 2009, H1N1 influenza A spread quickly across the United States (U.S.), infecting 43–89 million people nationwide and resulting in about 12,500 deaths [1, 2]. In 2014 and 2015, Ebola affected nine countries worldwide, infecting almost 23,000 individuals, killing more than 9000 and costing at least $1.6 billion or 5 % of the gross domestic product of the most heavily affected countries in West Africa [3, 4]. Early detection of bioterrorism, pandemics and outbreaks of emerging pathogens can minimize morbidity, mortality and costs, making it an essential component of health security [5].

Disease detection and monitoring is accomplished through surveillance, which involves the collection, analysis and interpretation of health-related data to inform and guide public health practice [6]. Traditionally, public health surveillance has been conducted using a combination of laboratory or clinical diagnostic data, which often involves under- and/or delayed reporting [2]. In contrast, syndromic surveillance involves collection of pre-diagnostic health criteria [7]. Though less targeted than traditional surveillance, syndromic surveillance is collected in near real-time, making it better able to rapidly identify suspicious events that may signal a bioterrorism attack or outbreak of an emerging pathogen [8, 9].

Various syndromic surveillance indicators have been collected and analyzed, including the number of ambulance dispatches, emergency department (ED) or ambulatory care clinic visits, cases of influenza-like illness and patients’ chief complaint, as well as indicators which may be associated with communicable disease syndromes, such as over-the-counter medicine and product sales [8, 10, 11]. Some public health departments have also collected school-based data, such as all-cause student absenteeism rates [12–17], gastrointestinal (GI) illness [13], and school nurse visits for influenza-like illness (ILI) [13, 18, 19], and compared it to other community pre-diagnostic criteria [8, 10, 13, 14, 20]. Research indicates that some school-based syndromic surveillance indicators may be as accurate as other surveillance data in early event identification [14, 16, 18, 19]. For example, student absenteeism and nurse visits for ILI have been correlated with the numbers of cases of ILI seen in local EDs or the community. However, other researchers have found that school data, especially all-cause absenteeism, is delayed at identifying outbreaks when used alone compared to ED data [13, 15, 20].

In the past, school syndromic surveillance has primarily been used to help inform school closure decisions [13, 17, 19, 21] and to augment existing data collected from EDs [13, 16, 18, 20] rather than used in isolation for early event identification. Some syndromic surveillance programs have been implemented solely on an ad hoc basis. For example, two school syndromic surveillance programs were reported as only being used during the 2009 H1N1 pandemic [16, 18]. Because school absenteeism data has the potential to inform both local policies and public health actions within communities, it is important to understand the current practices and attitudes towards syndromic surveillance in schools. The purpose of this study was to identify how many schools in one state are collecting and reporting syndromic surveillance data, and school nurses’ attitudes regarding this type of surveillance.

## Methods

This study was an adjunct to a larger assessment of Missouri school preparedness for biological events. The primary study involved a pre/post intervention design aimed at improving Missouri K-12 schools’ disaster preparedness during the 2013/2014 school year. The present study uses data from all participating schools, regardless of whether a pre or post-intervention survey was completed. If only one questionnaire was completed, that data was used for analysis (*n =* 86); if both a pre- and post-intervention questionnaire were completed (*n =* 47), only the post-intervention data was included in analysis. This resulted in a cross sectional dataset (*N* = 133). School nurses belonging to the Missouri Association of School Nurses (MASN) were emailed a link to an online questionnaire administered through Qualtrics®; the original and two follow-up recruitment emails were sent at the beginning and end of the 2013/2014 school year. Nurses who covered more than one school were asked to answer the survey questions from the perspective of the largest school they covered. The Saint Louis University Institutional Review Board approved this study.

### Instrument

The survey instrument used in a previous school preparedness study [22] was used as the basis for this questionnaire. In addition, questions were added to more thoroughly assess school-based syndromic surveillance. Psychometric testing conducted on the instrument has been described previously [22]. The questionnaire consisted of 18 items plus demographic questions. The following items were measured: a) whether the health department has approached the school about collecting syndromic surveillance data (1 item), b) if the school has discussed collecting syndromic surveillance data (1 item), c) attitudes towards syndromic surveillance (8 items), d) if the school nurse participated in the educational intervention specific to syndromic surveillance (2 items) and e) whether the school is collecting and/or reporting syndromic surveillance data (6 items).

### Data analysis

All analyses were conducted using Statistical Package for the Social Sciences (SPSS®) 22.0. Descriptive statistics were computed for each question. Z-Score tests were used to compare collection versus reporting of syndromic surveillance data. Chi squares were conducted to compare those schools approached by public health officials to assess the likelihood of collected and reported syndromic surveillance data. Multivariate logistic regressions were conducted to delineate factors associated with collection and reporting of syndromic surveillance indicators. Bivariate analyses were conducted prior to regression analyses; only variables significant in bivariate analysis were included in multivariate analyses. Only final models are reported. A critical p value of .05 was used for all analyses.

## Results

In total, 133 school nurses participated in the study (33.6 % response rate), with almost all nurses being female (99.2 %, *n* = 132) and white (95.5 %, *n* = 127). Almost half (48.9 %, *n* = 65) were 51–60 years old, and another quarter (23.3 %, *n* = 31) were 41–50. Most had a bachelor’s (39.1 %, *n* = 52) or associate’s (22.6 %, *n* = 30) degree. Respondents were almost equally divided between being a school nurse (41.4 %, *n* = 55) versus a lead nurse (58.6 %, *n* = 78); lead nurses are those who have administrative responsibility over other nurses. Approximately half (51.9 %, *n* = 69) reported participating in their school disaster planning committee. Nurses covered from 1 to 17 schools, with most (63.2 %, *n* = 84) working at only one. Of those who cover multiple schools (*n* = 49), almost half (44.9 %, *n* = 22) cover two schools. All grade levels and school sizes were represented, but most had 501 or more students (42.9 %, *n* = 57) or 300 or fewer (24.8 %, *n* = 33). Half (49.2 %, *n* = 65) were elementary schools, and the other half were almost equally divided between middle schools (22.7 %, *n* = 30) and high schools (28 %, *n* = 37). Almost all schools (94.7 %, *n* = 126) were public. The majority of schools were in a suburban (45.6 %, *n* = 61) or rural (41.4 %, *n* = 55) setting; only 12.7 % (*n* = 17) were urban.

### Collection versus reporting of school syndromic surveillance data

Half of the nurses (50.4 %, *n* = 67) indicated that their local health department had approached their school about collecting syndromic surveillance data. Nurses were asked whether their school collects and/or reports three syndromic surveillance indicators: student absenteeism, influenza-like illness (ILI) and gastrointestinal (GI) illness. Almost all schools (90.2 %, *n* = 120) are collecting at least one syndromic surveillance indicator. The most frequently reported indicators collected are absenteeism and ILI (72.2 and 65.4 %, respectively; Table 1). Schools are significantly less likely to collect GI illness (48.9 %, *n* = 65) compared to either absenteeism or ILI (*p* < .001 for both comparisons).

**Table 1:** Collection versus reporting of school syndromic surveillance indicators

Syndromic surveillance indicator	Data collected *N* = 133 % (n)	Data reported *N* = 133 % (n)	*p* value^a^
Any syndromic surveillance indicator	90.2 (120)	49.6 (66)	**< .001**
Absenteeism	72.2 (96)	15.8 (21)	**< .001**
Flu-like Illness	65.4 (87)	47.4 (63)	**< .001**
Gastrointestinal Illness	48.9 (65)	34.6 (46)	**< .05**

^a^ Determined by the Z-Score test

Schools are collecting more syndromic surveillance data than they are reporting to the local health department for all indicators (*p* < .001 for all comparisons; Table 1). Half of all schools (49.6 %, *n* = 66) report at least one syndromic surveillance indicator. The most frequently reported syndromic surveillance indicator is ILI (47.4 %, *n* = 63). Schools are significantly more likely to report ILI compared to GI illness (*X* ^2^ = 71.8, *p* < .001) or absenteeism (*X* ^2^ = 18.6, *p* < .001). Schools that were approached by public health officials were significantly more likely to collect (*X* ^2^ = 7.1, *p* < .01) or report (*X* ^2^ = 33.8, *p* < .001) any syndromic surveillance indicator compared to schools not approached by public health. The school nurses who reported that they are not currently collecting any syndromic surveillance data (*n* = 13) were asked if their school had discussed the possibility of collecting this data in the future. Almost all (92.3 %, *n* = 12) indicated that their school has not discussed future collection of syndromic surveillance data.

Determinants of school nurses’ collection of ILI, GI and absenteeism data included: perception that the local health department was interested in school-based syndromic surveillance data (ILI and GI), perceived administrative support for collection of syndromic surveillance data (ILI), knowledge of analyzing syndromic surveillance data (ILI and absenteeism), knowledge of collection of syndromic surveillance data (ILI), and perceived adequate resources for collection of syndromic surveillance data (GI; Table 2). The factor most strongly associated with increased reporting of syndromic surveillance data was the school nurses’ perception that the local health department was interested in collecting school syndromic surveillance data, which then increased the reporting of absenteeism (OR = 10.0 [CI = 2.8–35.9], *p* < .001), ILI (OR = 12.7 [CI = 4.7–34.5], *p* < .001), and GI Illness (OR = 9.2 [CI = 3.3–25.4], *p* < .001; Table 3). Other factors predicting school nurses’ reporting of ILI, GI and absenteeism data included the health department having approached the school about collection of syndromic surveillance data (ILI and GI), and the nurse being a member of the school disaster planning committee (ILI; Table 3).

**Table 2:** Factors related to school collection of student influenza-like illness, Gastrointestinal illness, and/or Absenteeism data

Variable	Influenza-like illness		Gastrointestinal illness		Absenteeism	
	OR (95 % CI)	*p*	OR (95 % CI)	*p*	OR (95 % CI)	*p*
Perception that health department is interested in collecting school syndromic surveillance data	16.6 (3.9–71.3)	**< .001**	4.7 (2.1–10.7)	**< .001**	NIM	NS
Administrators support syndromic surveillance data collection	9.5 (1.6–57.3)	**< .05**	NIM	NS	NIM	NS
Nurse knows how to analyze syndromic surveillance data	9.4 (1.4–63.5)	**< .05**	NIM	NS	2.5 (1.1–6.0)	**< .05**
Nurse knows how to collect syndromic surveillance data	4.8 (1.1–20.2)	**< .05**	NIM	NS	NIM	NS
School has resources to collect syndromic surveillance data	NIM	NS	2.7 (1.1–6.1)	**< .05**	NIM	NS

*OR = odds ratio; CI = Confidence interval; NS = Non-significant; NIM = Not in Model

**Table 3:** Factors related to school reporting of student influenza-like illness, Gastrointestinal illness, and/or Absenteeism data to the health department

Variable	Influenza-like illness		Gastrointestinal illness		Absenteeism	
	OR (95 % CI)	*p*	OR (95 % CI)	*p*	OR (95 % CI)	*p*
Perception that health department is interested in collecting school syndromic surveillance data	12.7 (4.7–34.5)	**< .001**	9.2 (3.3–25.4)	**< .001**	10.0 (2.8–35.9)	**< .001**
Health department has approached school about collecting syndromic surveillance data	3.1 (1.2–8.3)	**< .05**	4.6 (1.6–13.1)	**< .01**	NIM	NS
Nurse is a member of the school disaster planning committee	2.8 (1.1–7.1)	**< .05**	NIM	NS	NIM	NS

*OR = odds ratio; CI = Confidence interval; NS = Non-significant; NIM = Not in Model

### School nurses’ attitudes regarding syndromic surveillance

School nurse participants were asked eight attitudinal questions regarding syndromic surveillance. Most nurses (79.7 %, *n* = 106) agreed that collecting school-based syndromic surveillance data is important and that this data can help rapidly identify a biological event (75.9 %, *n* = 101; Table 4). Nurses were significantly more likely to indicate that they know how to collect syndromic surveillance data versus knowing how to analyze it (51.1 % vs. 24.4 %, *X* ^2^ = 47.4, *p* < .001). School nurses were significantly more likely to indicate that the school has the resources to collect syndromic surveillance data than having administrator support to collect such data (58.6 % versus 35.3 %, *X* ^2^ = 21.0, *p* < .001). However, when comparing lead nurses to non-lead nurses, non-lead nurses were more likely than lead nurses to indicate that their school has the resources to collect syndromic surveillance data (67.9 % vs. 45.5 %, *X* ^2^ = 6.7, *p* < .01; Table 4) and that they have time to collect syndromic surveillance data (76.9 % vs. 60.0 %, *X* ^2^ = 4.4, *p* < .05; Table 4).

**Table 4:** Lead nurses versus non-lead nurses’ attitudes regarding syndromic surveillance

Statement	All Nurses	Lead nurse vs. Not lead nurse		
	*N* = 133	*N* = 133		
		Not lead nurse *N* = 78	Lead nurse *N* = 55	Lead nurse vs. not lead nurse
	Strongly agreed or agreed % (n)	Strongly agreed or agreed % (n)	Strongly agreed or agreed % (n)	*p* value^a^
School syndromic surveillance data collection is important	79.7 (106)	80.8 (63)	78.2 (43)	NS
School syndromic surveillance data can aid in rapid identification of a biological event	75.9 (101)	75.6 (59)	76.4 (42)	NS
I have time to collect syndromic surveillance data	69.9 (93)	76.9 (60)	60.0 (33)	**< .05**
My school has the resources needed to collect syndromic surveillance data	58.6 (78)	67.9 (53)	45.5 (25)	**< .01**
I know how to collect syndromic surveillance data for my school	51.1 (68)	50.0 (39)	52.7 (29)	NS
Our local health department is interested in collecting school syndromic surveillance data	45.1 (60)	47.4 (37)	41.8 (23)	NS
My school administrators support syndromic surveillance data collection	35.3 (47)	35.9 (28)	34.5 (19)	NS
I know how to analyze syndromic surveillance data	29.3 (39)	24.4 (19)	36.4 (20)	NS

*NS* Non-significant

^a^Determined by the Chi-square or Fisher’s exact test (when cell size ≤ 5)

## Discussion

The early detection of infectious diseases within a community is a critical public health function, and an important element of health security. School settings can quickly amplify the rate of disease transmission, making early disease detection and monitoring particularly critical in order to prevent and mitigate community outbreaks. School-based syndromic surveillance has been shown to mirror the local community and ED disease surveillance data [14, 16, 18], as well as play a role in informing school closure decisions [13, 17, 19]. Therefore, school-based syndromic surveillance data can play an important role in community early detection programs and school disaster plans. In addition, school-based syndromic surveillance data has been used during past events to inform and develop risk communication messages for the public regarding status of an outbreak in the community [21]. In order to have this data available to enhance health security, schools must take an active role in both collecting and reporting syndromic surveillance data. The Missouri K-12 schools in this study demonstrated that school nurses can play a vital role in assisting with the collection and reporting of syndromic surveillance indicators, particularly since almost all (90 %) of the school nurses surveyed indicated that they were already collecting at least one syndromic surveillance indicator. Though almost all of the schools are collecting at least one syndromic surveillance indicator, only half of the nurses report this data to their local public health department. Fewer than a quarter of the schools report absenteeism data to public health, though almost three-quarters collect this data. The most plausible explanation for this is that schools collect absenteeism data for tracking truancy and obtaining state funding based on student enrollment rather than for the intention of using absenteeism as a syndromic surveillance indicator. It is also possible that school nurses do not have an incentive to report this data or understand the importance of syndromic surveillance data to community resilience. Regardless of the reason(s) why, it is worrisome that this valuable set of data is not routinely reported.

The lack of reporting for absenteeism is particularly concerning since research indicates that communicable disease transmission is common in school settings [12, 13, 16, 17] and that an increase in student absenteeism could be a precursor to, or signal a community-wide outbreak. In addition, children are often disproportionally affected during biological events [17], so it is appropriate to use absenteeism data to inform school closure decisions [17, 20]. For example, during the 2009 H1N1 influenza A pandemic, many schools across the country closed, citing absenteeism as their primary rationale along with high levels of illness [17]. Many of these schools had already invested substantial resources into their response and preparedness efforts for the H1N1 influenza A pandemic, yet the absenteeism data still prompted many school closures [17]. Therefore, it is especially important that schools routinely report their absenteeism data due to the potential impact it may have on school closure and other policy decisions in future communicable disease outbreaks. Challenges with utilizing school absenteeism data include its application to localized outbreaks, optimization of school closures, development and widespread acceptance of a single integrated system [12, 17, 20], so it important to recognize that school absenteeism alone should not inform school closure decisions.

This study found that public health official engagement with schools, or even the perception of their interest in collaboration, is associated with higher collection and reporting of school-based syndromic surveillance data. In this study, nurses’ perception of public health officials’ interest in school-based syndromic surveillance data was associated with higher collection of influenza-like and gastrointestinal illness indicators. Of course, data collection is only the first step in syndromic surveillance; reporting this data is vital to its use. A critical finding from this study is that school nurses who believed public health officials were interested in school-based data were 12.7, 10 and 9.2 times more likely to report influenza-like illness, gastrointestinal illness and absenteeism data, respectively, compared to nurses who did not share this belief. In addition, school nurses who reported that the health department had approached them about collecting syndromic surveillance data were 3.1 and 4.6 times more likely to report influenza-like and gastrointestinal illness data, respectively, compared to nurses who had not been approached by public health officials. The transition from the collection to reporting of syndromic surveillance indicators is clearly augmented when health departments express interest in the information by approaching the school.

An easy intervention to address this is for public health officials to engage school nurses and administrators in a dialogue about the role schools play in community health security. For instance, public health officials could point out that the Centers for Disease Control and Prevention (CDC) recommend in their pandemic planning checklist for K-12 schools [23] that schools consider engaging in community-wide syndromic surveillance programs. Findings from this study indicate that public health officials could increase their access to school-based syndromic surveillance data by simply letting the nurse or school administrators know that they are interested in this data. Given that so many schools are already collecting at least some types of syndromic surveillance data, it would not be a tremendous burden for the school nurse or administrator to simply report this data to local public health. Enhancing the partnership between schools and public health would also have other benefits for community health security and resilience by increasing coordination across the agencies, a gap that has been identified in multiple past studies [22, 24].

Other important predictors for the collection of syndromic surveillance indicators included nurses’ knowledge of how to collect and analyze syndromic surveillance data, and the perception that school administrators supported syndromic surveillance data collection. It is also notable that if a school nurse has served as a member of their school’s disaster planning committee, the reporting of influenza-like illness data increased. These factors are all actionable items that could greatly influence the level of collection and reporting of syndromic surveillance data by schools. Educational programs related to the collection and, more importantly, analysis of school-based syndromic surveillance data could be developed to aid nurses in gaining this knowledge. Half of the school nurses in this study self-identified as knowing how to collect syndromic surveillance data for their school, but less than a third indicated that they know how to analyze the data. Reporting of syndromic surveillance data to the health department is important, but internal review and analysis of the data would strengthen school resilience and increase the likelihood of early identification of an event; it could also be used to help inform school closure decisions during future biological events [17, 19]. If school-based syndromic surveillance educational programs were developed, it would be critical that they be offered face-to-face, such as a presentation at a national conference, as research has indicated that school nurses overwhelmingly prefer this format [25].

Another potential intervention to increase collection and reporting of school-based syndromic surveillance data would be to develop an educational program or biological event disaster exercise targeted at school administrators. Administrators determine the disaster planning priorities for their school, which could include syndromic surveillance participation. As of July, 2015, no existing studies could be found that describe school administrators’ attitudes, beliefs or priorities related to school biological event preparedness. Two studies have examined school administrators’ attitudes towards and involvement in school emergency management planning, but neither one specifically assessed biological events [26, 27]. One study measured school administrators’ perceptions of their school’s readiness for “all-hazards”, but biological events were not included in the disasters assessed [27]. The second study found that school administrators recognize their responsibility for school or district emergency management plans, either directly if they are on the planning committee or indirectly if they delegate that responsibility to others [26]. The Umoh [26] study assessed perceptions about administrators’ preparedness for terrorist threats, but biological events were not explicitly addressed; the study focused on bomb and active shooter threats. Based on the limited research found regarding school administrators and biological events, it is assumed that neither a needs assessment nor an intervention has been attempted to address these critically influential individuals. Future studies should assess school administrators’ knowledge and/or beliefs regarding the importance of school preparedness for bioterrorism, pandemics or outbreaks of emerging pathogens and the role that schools play in community resilience against these events. This could improve school participation in community syndromic surveillance programs, as findings from this study indicate that it is critical to gain school administrators’ support for the collection and analysis of school-based syndromic surveillance data.

Limitations of this study include the possibility of some self-selection bias by the subjects due to the study design. Also it is important to recognize that the study data was only collected in Missouri, and only from nurses belonging to the Missouri Association for School Nurses (MASN), which could limit some of the generalizability, particularly since not all school nurses in the state belong to MASN. Strengths of this study include the fact that the demographics of the nurse participants in this study are nearly identical to those of school nurses nationwide [28]. Additionally, school participation in the collection and reporting of syndromic surveillance data, and school nurses’ attitudes regarding syndromic surveillance have not been previously assessed. Future studies could further elucidate the factors influencing the implementation of syndromic surveillance programs in schools including both gathering as well as reporting of collected data.

## Conclusion

In this study of K-12 Missouri school nurses, it was found that school nurses are collecting syndromic surveillance indicators at a relatively high rate, yet less than half of this data gets reported to local public health officials. School nurses also strongly agree that syndromic surveillance data collection is important, and can aid in the swift identification of a biological attack. Conversely, school nurses do not generally perceive a high level of support or interest in syndromic surveillance data collection from their school administrators or the local health department. A pivotal finding in this study was that when the local health department showed an interest in syndromic surveillance data collection, both the collection and reporting of syndromic surveillance data dramatically increased. Therefore, it is important for local health departments and school administrators to discuss the collection and reporting of syndromic surveillance data with school nurses. This study provides valuable insight into factors influencing the decisions to implement syndromic surveillance programs in schools. Given that many schools are actively pursuing biological event and pandemic planning [22], this information should prove timely and beneficial to future planning efforts.
